# Germline Mutations in BRCA1 and BRCA2 in Breast Cancer Patients with High Genetic Risk in Turkish Population

**DOI:** 10.1155/2019/9645147

**Published:** 2019-01-01

**Authors:** Caglayan Geredeli, Nurgul Yasar, Abdullah Sakin

**Affiliations:** ^1^Okmeydani Training and Research Hospital, Medical Oncology Clinic, İstanbul, Turkey; ^2^Yuzuncu Yil University Medical School, Department of Medical Oncology, Van 65090, Turkey

## Abstract

**Background:**

The guidelines recommend considering the BRCA1 and BRCA2 germline mutations in female patients with breast carcinomas. In this retrospective study, the BRCA1/2 mutation prevalence in high-risk breast carcinoma patients in a Turkish population was investigated.

**Materials and Methods:**

In high genetic risk breast carcinoma patients, the BRCA1 and BRCA2 germline mutations were identified by applying next-generation sequencing.

**Results:**

The results showed BRCA1/2 mutations in 19% of the total patients. In those with first-degree relatives with breast carcinoma histories, the BRCA1/2 mutation prevalence was also 19%. In the patients younger than 40 years old, the BRCA1/2 mutation prevalence was 19.5%. In the triple-negative breast carcinoma patients younger than 60 years old, the BRCA1/2 mutation prevalence was 24.2%. In the patients younger than 40 years old with triple-negative breast carcinomas, BRCA1/2 mutation positivity was found in 37.5% of the patients. Overall, in the Turkish population, the BRCA1/2 mutation prevalence ranges from 19% to 37% in patients with high-risk breast carcinomas.

**Conclusion:**

It is recommended to check for BRCA1/2 mutations in all high-risk breast carcinoma patients in the Turkish population.

## 1. Introduction

In individuals with BRCA1 and BRCA2 mutations, the risk of breast and* ovarian* carcinoma development is very high. These are hereditary mutations [1, 2], and in people who have them, the risk of breast carcinoma development is 80%. The risk of ovarian carcinoma development in that population is 40% [1]. The breast carcinoma incidence increases in healthy women who have first-degree relatives with breast carcinomas. In particular, if the number of first-degree relatives with breast carcinomas is three or more, or if they are younger than 50 years old, the risk for developing a breast carcinoma is greatly increased [3].

In the healthy female population, the prevalence of BRCA mutation is around 2% [4] and in those with breast carcinoma histories in their families, the prevalence ranges from 9 to 21% [5–8]. In a Swedish study, BRCA1/2 mutations were found in 9% of the women who had first- or second-degree relatives with breast or ovarian carcinomas. The BRCA1 and BRCA2 mutations occurred in 6.8% and 2.1% of the patients, respectively [5]. In a study performed in China, the BRCA mutation prevalence in the breast carcinoma patients was 3.5%; however, the prevalence was 9.1% in the patients with risk factors for breast carcinomas [6, 9, 10]. In the USA, in those with breast carcinoma histories in their families, the BRCA1/2 mutation prevalence was 12% [7]. In one study of Korean women with breast carcinoma histories in their families, the BRCA mutation prevalence was 21.7% (BRCA1 = 9.3% and BRCA2 = 12.4%) [8]. The BRCA mutation prevalence was found to be around 60% in an Ashkenazi Jewish population with ovarian carcinomas. In the patients with breast carcinomas, the BRCA mutation prevalence was 30% and 10% in the patients younger and older than 40 years old, respectively. Even in an Ashkenazi Jewish population without breast and ovarian carcinoma histories in their families, the BRCA mutation prevalence was 10% [11]. In Israel, the BRCA mutation prevalence in patients with ovarian carcinomas was 30%; however, the prevalence was 45% in the ovarian carcinoma patients with family histories. In an Ashkenazi Jewish population in Israel, the BRCA mutation prevalence was 34%, and in those with family histories, this prevalence increased to 55% [12]. Although Israeli Jews and Arabs live in the same region, the BRCA mutation frequency was 5.6% in the women with breast carcinomas in the Arab community [13]. In the Palestinian Arabs living in the same region, the BRCA mutation prevalence was below the world average [14].

In young patients with breast carcinomas, the BRCA mutation prevalence is higher, ranging from 5.9 to 23% [8, 13, 15–18]. In the British population, in patients with breast carcinomas who are younger than 36 years old, the BRCA1/2 mutation prevalence was 5.9% (BRCA1 = 3.5% and BRCA2 = 2.4%) [15]. In the French population, in breast carcinoma patients who are younger than 46 years old, the BRCA1/2 mutation prevalence was 9.1% (BRCA1 = 6.5% and BRCA2 = 2.6%) [16]. In the USA, in a population of young women under 35 years old, the total BRCA1/2 mutation prevalence was 9.4% (BRCA1 = 5.9% and BRCA2 = 3.5%). The prevalence was 12% (BRCA1 = 7.1% and BRCA2 = 4.9%) in women under 45 years old [17]. In Korea, in young breast carcinoma patients under 35 years old with no family histories, the BRCA1/2 mutation prevalence was 10% [8]. In the Arab community, in patients younger than 40 years old with breast carcinomas, the BRCA mutation prevalence was 10.8%; however, the prevalence was 5.1% in women between 40 and 50 years old [13]. One carcinoma center found a BRCA1/2 mutation prevalence of 23% (BRCA1 = 17% and BRCA2 = 6%) in breast carcinoma patients under 35 years old [18].

The BRCA mutation prevalence in patients with triple-negative breast carcinomas is very high [19–23]. In the Canadian population, in triple-negative breast carcinoma patients under 40 years of age, the BRCA1/2 mutation prevalence was 11% [19]. In the Greek population, in triple-negative breast carcinoma patients, BRCA1/2 mutations were found in 16% of the patients [20]. In the German and Austrian community, in triple-negative breast carcinoma patients, the BRCA1/2 mutation prevalence was 21% [21]. In women with triple-negative breast carcinomas in the Hispanic population, the BRCA1/2 mutation prevalence was 23% [22]. In triple-negative breast carcinoma cases in China, the BRCA mutation prevalence was 25% [23]. In the USA, in women with triple-negative breast carcinomas, BRCA mutations were found in almost one-third of the patients [24]. Overall, BRCA1 mutations were more prevalent in the triple-negative breast carcinoma cases, while BRCA2 mutations were more prevalent in the hormone-positive tumors [25].

## 2. Materials and Methods

This was a retrospective single-center study performed in the Medical Oncology Clinic of the Okmeydani Education and Research Hospital in Istanbul. Istanbul is Turkey's largest city, with approximately 15 million people living there. The Okmeydani Education and Research Hospital is the largest state hospital in Istanbul. The study data was obtained by screening the clinical files of the breast carcinoma patients who were treated between April 2013 and February 2017, and whose BRCA mutations were investigated.

The characteristics of the patients with high genetic risks were taken from the patient files. The high genetic risk breast cancer patients included those under 40 years old, those with triple-negative breast cancer and younger than 60 years old, those with breast cancer histories in first-degree relatives, those with both breast and ovarian cancer, and those with bilateral breast cancer.

The histological tumor types and hormone receptor states were recorded. The estrogen receptor (ER), progesterone receptor (PR), and human epidermal growth factor receptor 2 (HER2) statuses were also recorded. The cases in which all of these were negative were considered to be triple-negative carcinomas. The BRCA1 and BRCA2 mutations were investigated using next-generation sequencing (NGS).

The BRCA1 (NG_005905.2 and NM_007294) and BRCA2 (NG_012772.3 and NM_000059) sequences from the National Center for Biotechnology Information (NCBI) database (http://www.ncbi.nlm.nih.gov) were used as reference sequences. The NGS sequencing data were analyzed using SeqPilot software (JSI Medical Systems, Ettenheim, Germany).

The Statistical Package for the Social Sciences (SPSS) version 15.0 for Windows was used for the statistical analysis. The number and percentage were used for the categorical variables, while the mean and standard deviation were used to define the numeric variables. The Mann-Whitney U test was used for the independent groups because of the nonparametric distribution of the variables, and the chi-squared analysis was used to compare the ratios for the independent variables. The Monte Carlo simulation was applied in those cases in which the conditions were not met. The statistical significance level of alpha was accepted as p<0.05.

## 3. Results

A total of 99 patients were included in this study, and their average age was 45.6 years old (range = 27–82 years). Sixty-eight patients were premenopausal (68%) and 31 patients were postmenopausal (31%). In 21 (21.2%) patients, there were breast carcinoma histories in their first-degree relatives, and in 8 patients there were breast carcinoma histories in their second-degree relatives. In terms of the tumor localization, the breast carcinomas were localized in the right breast in 47 (47%) patients and in the left breast in 45 patients (45%), and they were bilateral in 7 (7%) patients. When the histological tumor type was considered, there were invasive ductal carcinomas in 86 patients, invasive lobular carcinomas in 10 patients, and other types in 3 patients. Regarding the characteristics of the receptors, 60 patients were ER positive and 39 were ER negative. The PR was positive in 53 patients and negative in 46 patients. In 10 patients, the HER2 positivity was 3+. Triple-negative tumors were found in 33 patients (Table 1).

Twenty-one patients had first-degree relatives with breast carcinomas. Seven patients had bilateral breast carcinomas, and 7 patients had ovarian carcinomas in addition to breast carcinomas. Thirty-three of the patients under 60 years of age exhibited triple-negative features. Forty-one patients were under 40 years of age (Table 1).

BRCA1/2 positivity was found in a total of 19 patients (19%) (Table 2). Eleven patients (11%) had BRCA1 positivity and 8 patients (8%) had BRCA2 positivity. The average ages of the patients with and without BRCA1/2 positivity were 43.7 years old (range = 27–63 years) and 46.0 years old (range = 28–82 years), respectively (Table 2) (p value). Among the 21 patients with breast carcinoma histories in first-degree relatives, 4 (19%) patients had BRCA1/2 positivity. BRCA1 positivity was detected in 2 (9.5%) patients and BRCA2 positivity was detected in 2 (9.5%) patients (Table 2). Among the 8 patients with breast carcinomas in second-degree relatives, only 1 patient (12.5%) had a BRCA1 mutation (Table 2). Among the 7 patients with bilateral breast carcinomas, only 1 (14%) patient exhibited BRCA1/2 positivity. Among the 7 patients with both breast and ovarian carcinomas, only 3 patients (42.8%) exhibited BRCA1/2 positivity (Table 2). Two of those patients (28.5%) were BRCA1 positive and 1 (14.2%) patient was BRCA2 positive. Among the 33 patients under 60 years of age with triple-negative features, 8 (24.2%) had BRCA1/2 positivity. Among these patients, BRCA1 positivity was detected in 6 (18.1%) patients and BRCA2 positivity was detected in 2 (6.0%) patients (Table 2). In the patients under 40 years of age (n=41), 8 (19.5%) exhibited BRCA1/2 positivity. Among these patients, both the BRCA1 positivity and the BRCA2 positivity were detected in 4 patients (9.75%) (Table 2). Eight of the patients (8%) under 40 years of age showed triple negativity. Among these patients, 3 (37.5%) exhibited BRCA1/2 positivity, 2 (25%) showed BRCA1 positivity, and 1 (12.5%) showed BRCA2 positivity (Table 2).

Among the 60 patients with ER positivity, 9 (15%) exhibited BRCA1/2 positivity. Among the 39 patients with ER negativity, 10 (25.6%) exhibited BRCA1/2 positivity. Among the 60 patients with ER positivity, 4 (6.6%) were BRCA1 positive, while 5 (8.33%) were BRCA2 positive. There was BRCA1 positivity in 7 (17.9%) of the 39 ER negative patients, while there was BRCA2 positivity in 3 (7.6%) of the patients. Among the 53 PR positive patients, 8 (15%) had BRCA1/2 positivity. Among the 46 patients with PR negativity, 11 (23.9%) had BRCA1/2 positivity. Among the 53 patients with PR positivity, positivity in both BRCA1 and BRCA2 was detected in 4 patients (7.5%) for each mutation. Among the 46 PR negative patients, the BRCA1 and BRCA2 were positive in 7 patients (15.2 %) and 4 patients (8.6%), respectively. Among a total of 99 patients, the HER2 was 3+ in 10 patients; however, none exhibited BRCA1/2 positivity.

In those patients with right breast carcinomas, the BRCA1 and BRCA2 were positive in 8 patients (72.7%) and 3 patients (37.5%), respectively. Among the 8 patients with left breast carcinomas, the BRCA1 and BRCA2 were positive in 3 patients (27.2%) and 5 patients (62.5%), respectively.

Regarding the 11 patients with BRCA1 mutations, 8 patients (72.9%) were in the premenopausal period and 3 patients (27.2%) were in the postmenopausal period. Among the 8 patients with BRCA2 mutations, 6 patients (25%) were in the premenopausal period and 2 patients were in the postmenopausal period.

With regard to the histological tumor subtypes, of the 86 with invasive ductal carcinomas, 11 patients exhibited BRCA1 positivity and 6 patients exhibited BRCA2 positivity. Among the 10 patients with invasive lobular carcinomas, BRCA2 positivity was detected in only 1 patient. In the other histological subtypes, only 1 patient exhibited BRCA2 positivity.

The age difference between the patients with concomitant breast and ovarian cancer (59.3 years old) and the other patients with breast cancer (44.5 years old) was statistically significant (p=0.007). That is, breast and ovarian carcinomas tended to coexist in the older ages. Five of the 7 patients were in the postmenopausal period, and 2 patients were in the premenopausal period; this difference was statistically significant (p=0.02). This finding showed that the patients with concomitant breast and ovarian carcinomas were frequently in the postmenopausal period.

In the patients under 60 years of age with triple-negative tumors, the tumor was in the right breast in 12 patients and in the left breast in 21 patients. The triple-negative cases were detected significantly more frequently in the left breast (p=0.01). The mean Ki-67 value (52%) in the triple-negative patients was significantly higher than the mean Ki-67 value (30%) in the other patients (p=0.002).

The average age of the 41 patients younger than 40 years old was 35.7 years old, and 97.6% of these patients were in the premenopausal period. The frequency of a breast carcinoma history in a first-degree relative of these patients under 40 years of age was statistically significantly higher than in the other patients (p=0.024).

The mean age of the patients with bilateral breast carcinomas (51.3 years old) was significantly higher than the mean age of the patients (44.8 years old) without bilateral breast carcinomas (p=0.025). All 7 patients with bilateral breast carcinomas were HER2-negative (p=0.045).

A total of 14 mutation types were identified in the BRCA mutation-positive patients, and the names of the mutations are given in Table 3.

## 4. Discussion

In previous population-based studies, the BRCA1/2 prevalence was found to be around 2–3.5% [6, 7]. However, the BRCA1/2 mutation prevalence is around 10% in the Ashkenazi Jewish population without a relative with a breast or ovarian carcinoma [11]. The BRCA1/2 mutation prevalence is higher in young patients, patients with first-degree relatives with breast or ovarian carcinomas, patients with triple-negative carcinomas, patients with bilateral carcinomas, patients with concomitant breast and ovarian carcinomas, and high genetic risk patients [5, 8, 12, 15, 17, 19–21, 24]. In most studies, the mutation rates were evaluated separately in the subgroups in which the risks of BRCA1/2 mutations were high. To our knowledge, in no study has the mutation prevalence been given based on all of these high-risk factors. In our study, we took into account all of these risk factors, and we evaluated them collectively by investigating the BRCA1/2 mutation prevalence in the patients with these risk factors. In these high-risk patients, we determined a total BRCA1/2 mutation prevalence of 19%, with a BRCA1 mutation prevalence of 11% and BRCA2 mutation prevalence of 8%.

Walsh et al. found a BRCA1/2 prevalence of 11% in those individuals with breast carcinoma histories in their families [7]. In Sweden, Loman et al. found a BRCA1/2 prevalence of 9% in those individuals with breast and ovarian carcinoma histories in their families [5]. In Korea, Han et al. found a BRCA1/2 mutation prevalence of 21.7% in those individuals with breast carcinoma family histories [8]. In a study from Israel, the BRCA mutation prevalence in patients with ovarian carcinomas was 30%, with a rate increase to 45% in those patients with family histories [26]. In the Ashkenazi Jewish population in Israel, the mutation prevalence was 34%, which increased to 55% in those having family histories [11, 12]. In a study of the Azerbaijani Turkish population living in Iran, Nahid et al. found a BRCA2 mutation prevalence of 10% in breast carcinoma patients [27]. Moreover, the BRCA1/2 mutation prevalence in the Turkish population was reported as 14% [28]. In our study, the BRCA1/2 positivity was 19% in the breast carcinoma patients with first-degree relatives having breast carcinoma histories. In these patients, the BRCA1 positivity was 9.5% and the BRCA2 positivity was 9.5%. In the patients with breast carcinoma histories in their second-degree relatives, the BRCA1 mutation prevalence was 12.5%. This prevalence (19%) was higher than the prevalence of 9–11% found in Europe and America, but similar to the 21% found in Asia. Although the Turkish and Israeli populations are in neighboring countries in the middle of Asia, the BRCA1/2 positivity was 19% in the Turkish population with breast carcinoma family histories, which was quite low when compared to the BRCA1/2 positivity prevalence of 45% seen in those with positive family histories in Israel.

The BRCA1/2 mutation prevalence in women with breast carcinomas is higher in patients under 40 years of age [8, 13, 15–18]. Malone et al., in their study conducted in the USA, found a BRCA1/2 mutation prevalence of 12% in women under 45 years of age [17]. Bayraktar et al. found BRCA1/2 mutations in 23% of the breast carcinoma patients under 35 years of age, according to the Anderson Carcinoma Center data [18]. Peto et al. found a BRCA1/2 mutation positivity prevalence of 5.6% in women with breast carcinomas under the age of 36 years old in the UK [15]. In France, Bonadona et al. found a BRCA1/2 positivity prevalence of 9.1% in women with breast carcinomas under the age of 46 years old [16]. El Saghir et al. found a BRCA1/2 mutation prevalence of 10.8% in patients with breast carcinomas under the age of 40 years old in an Arabic population [13]. In Korea, Han et al. found a BRCA1/2 mutation prevalence of 10% in young breast carcinoma patients under the age of 35 years old [8]. In our study, we found a BRCA1/2 mutation prevalence of 19.5% (BRCA1 = 9.75% and BRCA2 = 9.75%) in breast carcinoma patients under the age of 40 years old. This prevalence of 19.5% seems to be about double the BRCA1/2 mutation prevalence in the literature mentioned above. However, the prevalence found in this study was not found in a population-based study, and our prevalence rate belongs to a population with high-risk factors. Thus, in patients with breast carcinomas who are younger than 40 years old with genetic risk factors, the BRCA1/2 mutation prevalence was 19.5%.

Another subgroup in which BRCA1/2 mutations are frequently seen is women with triple-negative breast carcinomas [19–23]. In Germany and Austria, Muendlein et al. found a BRCA1/2 mutation prevalence of 21% in women with breast carcinomas showing triple-negative features [21]. Fostira et al. found a BRCA1/2 mutation prevalence of 16% in a Greek population of women with breast carcinomas showing triple-negative features [20]. In a Hispanic population, Villarreal et al. found a BRCA1/2 mutation prevalence of 23% in women with breast carcinomas showing triple-negative features [22, 29]. Li et al. found a BRCA1/2 mutation prevalence of 25% in women with breast carcinomas showing triple-negative features in a Chinese population [23]. In our study, we found a BRCA1/2 mutation prevalence of 24.2% in the patients with breast carcinomas showing triple-negative features. Our prevalence shows similarities with the above-mentioned prevalence of 20–25% registered in the literature. Overall, BRCA1/2 mutations are frequently seen in young women and women with breast carcinomas showing triple-negative features. In our study, there was a prevalence of 37.5% in the women with breast carcinomas showing triple-negative features under the age of 40 years old.

Krammer et al. found mostly BRCA1 mutations in those patients who showed triple-negative features and mostly BRCA2 mutations in the hormone-positive patients [25]. In our study, the BRCA1 and BRCA2 positivity prevalence was 6.6% and 8.3% in the patients with ER positivity, respectively. The BRCA1 and BRCA2 positivity prevalence was 17.9% and 7.6% in those patients with ER negativity, respectively. Our findings contribute to the research of Krammer et al. who argued that the BRCA1 mutation is more frequently seen in triple-negative patients.

## 5. Conclusion

In conclusion, in the Turkish population, the total BRCA1/2 mutation positivity prevalence was 19% in the patients with high-risk breast carcinomas. The BRCA1/2 mutation positivity prevalence was also 19% in the patients with breast carcinoma histories in their first-degree relatives. In the patients under 40 years old, the BRCA1/2 mutation positivity prevalence was 19.5%, and in the patients under 60 years old with triple-negative breast carcinomas, the BRCA1/2 mutation positivity prevalence was 24.2%. In the women with breast carcinomas showing triple-negative features under the age of 40 years old, the BRCA1/2 mutation prevalence was 37.5%. This shows that, as in other Turkish populations, the BRCA1/2 mutation frequency in women with high-risk breast carcinomas increases from 19% to 37%. In the Turkish population, all high-risk breast carcinoma patients should absolutely be evaluated in terms of BRCA1/2 mutations.

## Figures and Tables

**Table 1:** The characteristics of the patients.

Characteristics		**n**	%
Menopause Status	Premenopause	68	68
	Postmenopause	31	31

Family History	First-degree relative	21	21.2
	Second-degree relative	8	8.08

Tumor Localization	Right	47	47
	Left	45	45
	Bilateral	7	7

Tumor Histology	Invasive ductal carcinoma	86	86
	Invasive lobular carcinoma	10	10
	Others	3	3

Receptor Status	ER positive	60	60
	ER negative	39	39
	PR positive	53	53
	PR negative	46	46
	HER2 positive	10	10
	HER2 negative	89	89
	Triple-negative	33	33

High Risk Factors	First-degree relative	21	21
	Bilateral carcinoma	7	7
	Breast + Ovarian carcinoma	7	7
	Triple-negative	33	33
	Below 40 years old	41	41

**Table 2:** The characteristics of the groups.

**Characteristics**	**n**	**BRCA1/BRCA2**		**BRCA1**			**BRCA2**		
		**Negative**	**Positive**	**Negative**	**Positive**	**p**	**Negative**	**Positive**	**p**
Age [mean (min-max)]		46 (28-82)	43.7 (27-63)	45.7	45.5	0.781	46.0	41.4	0.190
Germline mutation		81 (81%)	19 (19%)	88 (88%)	11 (11%)		91 (91%)	8 (8%)	
Family History	21	17 (81%)	4 (19%)	19 (90.5%)	2 (9.5%)	1.000	19 (90.5%)	2 (9. 5%)	1.000
Bilateral breast carcinoma	7	6 (86%)	1 (14%)	7	0	0.351	6 (86%)	1 (14%)	1.000
Breast + ovarian carcinoma	7	4 (67.2%)	3 (42.8%)	5 (71.5%)	2 (28.5%)	0.173	6 (85.8%)	1 (14.2%)	0.456
Triple-negative tumor	33	25 (75.7%)	8 (24.2%)	27 (81.8%)	6 (18.1%)	0.172	31 (93.9%)	2 (6.0%)	0.715
Below 40 years old	41	33 (80.4%)	8 (19.5%)	37 (90.2%)	4 (9.5%)	1.000	37 (90.2%)	4 (9.5%)	0.715
Triple negative + below 40 years old	8	5 (62.5%)	3 (37.5%)	6 (75%)	2 (25%)		7 (87.5%)	1 (12.5%)	
ER Positive	60	51 (85%)	9 (15%)	56 (93.3%)	4 (6.6%)	0.316	55 (91.6%)	5 (8.33%)	0.824
ER Negative	39	29 (74.3%)	10 (25%)	32 (82%)	7 (17.9%)		36 (92.3%)	3 (7.6%)	
PR Positive	53	45 (84.9%)	8 (15%)	49 (92.4%)	4 (7.5%)	0.526	49 (92.4%)	4 (7.5%)	1.000
PR Negative	46	35 (76%)	11 (23.9%)	39 (84.7%)	7 (15.2%)		42 (91.3%)	4 (8.6%)	
HER 2 Positive	10	10 (100%)	0	10 (100%)	0	0.650	10 (100%)	0	1.000
Right Breast	47	36 (76.5%)	11 (23.4%)	39 (82.9%)	8 (17%)	0.262	44 (93.6%)	3 (6.3%)	0.710
Left Breast	45	37 (82.2%)	8 (17.7%)	42 (93.3%)	3 (6.6%)		40 (88.8%)	5 (11.1%)	
Invasive Ductal Carcinoma	86	69 (80%)	17 (19%)	75 (87%)	11 (12%)	0.351	80 (93%)	6 (6.9%)	0.245
Invasive Lobular Carcinoma	10	9 (90%)	1 (10%)	10	0	0.596	9 (90%)	1 (10%)	0.583

**Table 3:** Characteristics of BRCA positive patients.

**Patients**	**Age**	**ER**	**PR**	**CerbB2**	**1st degree relative breast cancer**	**Bilateral breast cancer**	**Both ** **ovarian** ** and breast cancers**	**BRCA1 mutation**	**BRCA 2 mutation**	**BRCA mutations found**
4.	34	negative	negative	negative	-	-	-	**positive**	-	p.R1203 (c.3607C>T)
8.h.g	38	negative	negative	negative	-	-	-	**positive**	-	p.S1613G (c.4837A>G)
10.r.i	36	positive	positive	negative	-	-	-	-	**positive**	c.9413dupT
11.s.ö	40	negative	negative	negative	**Positive**	-	-	**positive**	-	p.R1203 (c.3607c>T)
12.n.t	60	negative	negative	negative	**Positive**	-	**positive**	-	**positive**	IVS2 +1G>A (c.67+1G>A)
13.e.e	63	negative	negative	negative	-	-	**positive**	-	**positive**	IVS2 +1G>A (c.67+1G>A)
15.n.a	38	negative	negative	negative	-	-	-	-	**positive**	c.9413dupT
21.h. s	47	negative	negative	negative	-	-	-	**positive**	-	IVS18 +66G>A
										(c.5152+66g>A)
26.u.d	53	negative	negative	negative	-	-	-	**positive**	-	p.Q356 (c1067A>G)
27.m.a	54	negative	negative	negative	-	-	-	**positive**	-	p.S1613G(c.4837A>G
44.e.e	43	negative	negative	negative	**Positive**	-	-	-	**positive**	c.771_775deTCAA
49.g.t	51	positive	positive	negative	**Positive**	-	-	**positive**	-	IVS7 -34C>T (c.442-34C>T)
59.d.a	50	positive	positive		-	-	**positive**	**positive**	-	p.W1815 (c.5444G>A)
61.b.b	54	positive	positive	Negative	-	-	**positive**	**positive**	-	p.L1209lfs 10 (c.3624dupA)
63.f.s	41	negative	negative	negative	-	-	**positive**	**positive**	-	p.W1815 (c.5444G>A)
64.z.e	27	positive	positive	negative	-	-	-	-	**positive**	c.7487dupA
68.n.k	31	positive	positive	negative	-	-	-	-	**positive**	p.S1106R (c.3318C>G)
69.ç.g	32	positive	negative	negative	-	-	-	-	**positive**	P.W3106X(c.9317G>A)
85.d.t	38	positive	positive	negative	-	**positive**	-	**positive**	-	p.L1209lfs 10 (c.3624dupA)

## Data Availability

The data used to support the findings of this study are available from the corresponding author upon request.
